# Clinical profile of children and adolescents with autism spectrum disorder in Durban, South Africa

**DOI:** 10.4102/sajpsychiatry.v30i0.2230

**Published:** 2024-05-14

**Authors:** Manisharani Gangai, Enver Karim, Saeeda Paruk

**Affiliations:** 1 Department of Psychiatry, Faculty of Health Sciences, University of KwaZulu-Natal, Durban, South Africa

**Keywords:** autism spectrum disorder, clinical features, children and adolescents, South Africa, Durban, sociodemographic profile

## Abstract

**Background:**

There are often delays in accessing care and diagnosing autism spectrum disorders (ASDs), with little data from Southern Africa on the clinical profile of affected children and adolescents.

**Aim:**

To describe the socio-demographic and clinical variables of children and adolescents with ASD attending psychiatric services at two state hospitals in eThekwini Municipality, KwaZulu-Natal province, South Africa.

**Setting:**

Two state hospitals in KwaZulu-Natal province, South Africa.

**Methods:**

The retrospective chart review examined patient records for the period 01 January 2018 to 31 December 2021. Data were collated using a structured data questionnaire on birth and family history, current presentation, comorbid conditions, medications, and non-pharmacological interventions.

**Results:**

Of the 67 children and adolescents accessing care for ASD during the study period (including the coronavirus disease 2019 [COVID-19] pandemic lockdown period), most were males (89%), with a mean age standard deviation (s.d.) of 10.69 (s.d. 2.64) years. There was a delay between recognition of first symptoms and an ASD diagnosis of approximately three years. The most common reasons for referral were behavioural problems and speech delay, with 57 patients having delayed milestones (85%). Comorbid attention deficit hyperactivity disorder was reported in 55.2% (*n* = 37) of the patients and intellectual disability in 50.7% (*n* = 34), and the commonest comorbid medical condition was epilepsy (*n* = 20; 29.8%). All participants were on psychotropic medications, with 40 (59%) being on more than one agent.

**Conclusion:**

The delay in diagnosing ASD, high rates of comorbidity, and need for polypharmacy are concerning.

**Contribution:**

The study highlights the need for greater awareness of ASD in communities and health care workers to expedite diagnosis and facilitate prompt psychosocial support and rehabilitation.

## Introduction

Autism spectrum disorder (ASD) is a neuro-developmental disorder that is characterised by impairments in communication skills, preoccupation with restricted interests, and the demonstration of stereotyped behaviours and activities.^1^ This heterogenous disorder is a prominent cause of disability in young children, and social, educational, occupational and economic disadvantages can accumulate with development. The individual may present with variable mix of behavioural symptoms and be affected along a continuum of severity.^2^ Autism spectrum disorder encompasses intrinsic cognitive symptoms and is frequently associated with comorbid disorders.^3^

Historically grouped under ‘pervasive developmental disorders (PDDs)’ in the Diagnostic and Statistical Manual of Mental Disorders III (DSM–III) for its broad social and communication deficits, the term ASD is now used in the DSM–5 and International Classification of Diseases 11 (ICD–11) with clinical specifiers and modifiers for individual diagnostic differentiation.

The condition has long-term effects on the wellbeing and functioning of the individual and that of their family, with associated societal and economic costs, specifically related to their need for life-long care.^4^ The lifespan societal cost of ASD in high-income countries is estimated to be up to 2.4 million dollars per individual in the United States of America (USA)^5^, with the collective cost for all those affected being 32 billion pounds annually in the United Kingdom (UK),^6^ making the condition a public health priority.^7^ The costs associated with ASD in low to middle-income countries, such as South Africa, have not been determined or researched, making it difficult to establish the costs for the family and the health care system.

Autism spectrum disorder may be diagnosed by 18–24 months of age, with early signs being identified as early as 12 months of age.^8^ Notably, ASD occurs more frequently in males, with a male to female ratio of approximately 4.2:1.^9^ Early identification of ASD is critical because implementing early interventions can lead to significantly improved outcomes.^10^ There are multiple medical disorders that are often comorbid with ASD, such as epilepsy, food intolerance, gastrointestinal dysfunction, and psychiatric disorders including attention deficit hyperactivity disorder (ADHD), tic, anxiety, depression, sleep and behavioural problems such as aggressive and self-injurious behaviours.^11^

These co-occurring conditions can exacerbate the challenges faced by individuals with ASD, making management and treatment more complex.^12^ For instance, ADHD is a common comorbidity that can affect between 26% and 65% of individuals with ASD.^13^ Comorbid ADHD symptoms can worsen outcomes^14^ and delay detection of ASD.^15^ Anxiety disorders are also prevalent, affecting around 40% of those with ASD,^16^ with specific phobia and generalised anxiety disorder being the most common.^17^ Additionally, people with ASD are at higher risk of depression, suicidal ideation and suicidal behaviours, often stemming from social isolation.^18^ Depression may present with typical symptoms such as tearfulness, and self-harm,^19^ but also through ASD-specific symptoms such as irritability, or changes in behaviour patterns.^20^ Tic disorders, including Tourette syndrome and other tic behaviours, are more common in individuals with ASD than in the general population, with a prevalence rate of about 28% – 34%.^21,22,23^ The presence of tic disorders in individuals with ASD may also be linked to higher intelligence quotient (IQ) and more severe ASD and comorbid symptoms.^24^

As these comorbid disorders have a negative impact on the quality of life of the individual and their family, early intervention is aimed at decreasing disability and addressing comorbid conditions which may impact on prognosis.^25^ This study therefore aimed at describing the socio-demographic and clinical features including age of presentation and potential delay in diagnosis of children and adolescents affected by ASD attending public sector child psychiatric services in eThekwini Municipality of KwaZulu-Natal province, South Africa.

## Materials and methods

### Study design, site and sample population

A retrospective descriptive quantitative chart review study of children and adolescents diagnosed with ASD was conducted at the specialist psychiatry outpatient departments of the two hospitals for the period, 01 January 2018 to 31 December 2021 using the hospital register. The study included all patients who were 18 years or younger, and clinically fulfilled DSM–5 criteria for ASD. Both hospitals have a child and adolescent psychiatrist supervising the assessment and management of the patients. They also have access to clinical psychology, occupational therapy, social work, and physiotherapy services at the units.

### Measures

A structured socio-demographic and clinical questionnaire was designed to extract data from the clinical records, including age at presentation, gender, educational level, referral source, and if living in urban or rural area. Clinical information was collated on birth history, educational needs, family history, clinical presentation, developmental features (milestones), comorbid conditions, medications and non-pharmacological interventions.

### Statistical analysis

Data were collected by a senior psychiatry registrar and anonymised and analysed with Statistical Package for the Social Sciences (SPSS) version 28.0 (IBM Corp, Armonk, NY, USA) and Stata version 16.0 (StataCorp, College Station, TX, USA). Categorical data (e.g., age and educational level) were presented as frequencies and percentages, with descriptive statistics (mean and standard deviation [s.d.]) being used to describe the continuous data (e.g., gender) that were collected.

### Ethical considerations

The study was approved by the Biomedical Research Ethics Committee of the University of KwaZulu-Natal (BREC/00004009/2022), and permission to conduct it in the provincial hospitals was given by the KwaZulu-Natal Department of Health. The two hospital managers gave the consent for the records to be accessed.

## Results

### Demographic variables

During the 4 years in which the records were reviewed for patients who met the inclusion criteria, 67 files were identified for analysis and their data were extracted. There were significantly more males (*n* = 60, 89.6%) than females (*n* = 7, 10.4%) (*p* < 0.001), almost half (*n* = 32, 47.8%) were African, and the largest age group was 6–12 years old (Table 1). The majority (*n* = 66, 98.5%) of the patients resided in urban areas, slightly more than half (*n* = 37, 55.2%) were living with both parents, with the majority (*n* = 47, 70.1%) attending remedial or special educational needs schools. The 15 (22.4%) patients who were not schooling were either awaiting placement at special educational needs school, or their severe aggressive behaviour precluded admission currently to a school.

**TABLE 1:** Socio-demographic characteristics of children/adolescents with autism spectrum disorder (*N* = 67).

Variable	Characteristic	Number	Percent
Gender	Male	60	89.6
	Female	7	10.4
Race	African	32	47.8
	Indian	19	28.4
	Coloured	8	11.9
	White	8	11.9
Age (in years)	0–5	16	23.9
	6–12	27	40.2
	13–17	24	35.9
Social background	Urban	66	98.5
	Semi-rural	1	1.5
Living arrangements	Living with both parents	37	55.2
	Living with mother	21	31.3
	Living with member of extended family	6	9.0
	Foster care or children’s home	3	4.5
Current schooling type	Not currently schooling/preschool	15	22.4
	Remedial/special needs	47	70.1
	Mainstream	4	6.0
	Unknown	1	1.5

### Clinical variables

The mean and s.d. for the current age of the child (in years) was 10.69 (s.d. 2.64) years (Table 2), while the median and interquartile range (IQR) was 10 (9.0–12.0) years. The mean age of first symptom recognition by parent/caregiver was 4.10 years (s.d. 2.20), referral to psychiatry was 6.16 years (s.d. 3.79), and at first diagnosis of ASD was 7.38 years (s.d. 3.93). Help-seeking was mainly at paediatric medical services (*n* = 28, 41.8%) followed by general medical services (*n* = 22, 32.8%), with the main referrals being from mental health care practitioners (*n* = 20, 29.9%), paediatrics (*n* = 21, 31.3%), and other doctors (*n* = 22, 32.8%). The main reason for referral was behavioural problems (*n* = 61, 91.0%). In addition, 49 (73.1%) patients indicated that there was no history of mental illness in first degree relatives.

**TABLE 2a:** Clinical variables of children and adolescents with autism spectrum disorder.

Age	Mean	Standard deviation
Current age (in years)	10.69	2.64
Age of first symptom recognition (in years)	4.10	2.20
Age of ASD diagnosis (in years)	7.38	3.93
Age of referral to psychiatry (in years)	6.16	3.79

ASD, autism spectrum disorder.

**TABLE 2b:** Clinical variables of children and adolescents with autism spectrum disorder.

Variable	Characteristic	Number	Per cent
Help seeking	Teacher	2	3.0
	Medical	22	32.8
	Paediatrician	28	41.8
	Other for example Psychology/Occupational therapy/Speech therapy	15	22.4
Referral source	Mental health care practitioners	20	29.9
	Paediatrics	21	31.3
	Other doctors	22	32.8
	Speech therapy	1	1.5
	Social worker	3	4.5
Reason for referral	Scholastic problems	5	7.5
	Behaviour problems	61	91.0
	Speech delay	14	20.9
	Grant Application	1	1.5
	Hyperactivity	1	1.5
	Sleep Problems	1	1.5
	Suicidal ideation	1	1.5
Family history of mental illness in first degree relative	No	49	73.1
	Yes	15	22.4
	Unknown	3	4.5
Specify relationship of mentally ill family member	Mother	4	26.7
	Father	6	40.0
	Sibling	5	33.3

In assessing the clinical variables of the ASD, the majority of patients living with ASD were noted to have severity level 2 (*n* = 27, 40.3%), with similar numbers being non-verbal (*n* = 22, 32.8%) having limited speech (*n* = 23, 34.3%) and normal speech (*n* = 22, 32.8%) (Table 3). Three quarters (*n* = 50, 74.6%) had language delays, all lacked social interaction skills.

**TABLE 3a:** Autism spectrum disorder characteristics (*N* = 67).

Variables	Level 1		Level 2		Level 3	
	*n*	%	*n*	%	*n*	%
Severity of ASD	19	28.4	27	40.3	21	32.3

ASD, Autism spectrum disorder.

**TABLE 3b:** Autism spectrum disorder characteristics (*N* = 67).

Variables	Non-verbal		Some words		Normal speech	
	*n*	%	*n*	%	*n*	%
Speech	22	32.8	23	34.3	22	32.8

**TABLE 3c:** Autism spectrum disorder characteristics (*N* = 67).

Variables	No		Yes		Not recorded	
	*n*	%	*n*	%	*n*	%
Language delay	3	4.5	50	74.6	14	20.9
Lack of reciprocal social interaction	0	0.0	67	100.0	-	
Lack of pretend play	8	11.9	34	50.7	25	37.3
Head banging	8	11.9	36	57.3	23	34.3
Tantrums	6	9.0	41	61.2	20	29.9
Stereotypic movements	5	7.5	57	85.1	5	7.5
Hyperactivity	20	29.9	46	68.7	1	1.5
Aggression	27	40.3	34	50.7	6	9.0
Head size-microcephaly	66	98.5	1	1.5	-	-

**TABLE 3d:** Autism spectrum disorder characteristics (*N* = 67).

Variables	Normal		Abnormal		Not done	
	*n*	%	*n*	%	*n*	%
Electro-encephalography	35	52.2	20	29.9	12	17.9
Educational Psychology Assessment	2	3.0	50	74.6	15	22.4
Occupational Therapy Assessment	3	4.5	45	67.2	19	28.4
Visual screen	40	59.7	7	10.4	20	29.9
Hearing screen	43	64.2	5	7.5	19	28.4

**TABLE 3e:** Autism spectrum disorder characteristics (*N* = 67).

Variables	No		Yes		Unknown	
	*n*	%	*n*	%	*n*	%
Seizures in first year	32	47.7	4	6.0	31	46.3
Delayed milestones	7	10.4	57	85.0	3	4.6
**Obstetric and other history**						
Low birth weight	51	76.1	4	6.0	12	17.9
Prematurity	51	76.2	9	13.4	7	10.4
Paternal age > 40 at conception	3	4.5	0	0.0	64	95.5
Maternal age of > 35 years	5	7.4	2	3.0	60	89.6

In terms of comorbid disorders, 53 (79.2%) patients had an additional psychiatry diagnosis, with 37 (55.2%) having comorbid ADHD (Table 4). The prevalence of intellectual disability was approximately 50%.

**TABLE 4:** Comorbid psychiatric and medical disorders (*N* = 67).

Characteristic	No		Yes	
	*n*	%	*n*	%
Co-morbid psychiatry diagnosis/es	14	20.9	53	79.1
ADHD	30	44.8	37†	55.2
Intellectual Disability	33	49.3	34	50.7
HIV status – all negative	67	100.0	0	0.0
Asthma	66	98.5	1	1.5
Sinusitis	61	91.0	6	9.0
Bedwetting	59	88.1	8	11.9
Epilepsy	47	70.1	20	29.9

ADHD, attention deficit hyperactivity disorder; HIV, human immunodeficiency virus.

† , 32 males and 5 females.

One quarter (*n* = 25, 37.3%) of the patients were being assisted by a speech therapist, only seven (10.4%) by a social worker, and eight (11.9%) by a clinical psychologist. All the patients were on psychotropic medication, with 36 (53.7%) being on risperidone, 40 (59.7%) being on an anticonvulsant, and 40 (59.7%) being on more than one psychotropic drug (Table 5).

**TABLE 5:** Management strategies used for children and adolescents with autism spectrum disorder.

Variables	No		Yes		One		More than one	
	*n*	%	*n*	%	*n*	%	*n*	%
**Strategies**								
Speech therapy	42	62.7	25	37.3	-	-	-	-
Social worker	60	89.6	7	10.4	-	-	-	-
Clinical psychologist	59	88.1	8	11.9	-	-	-	-
Psychiatric meds	0	0.0	67	100.0	-	-	-	-
**Type of Medication**					-	-	-	-
Methylphenidate	48	71.6	19	28.4	-	-	-	-
Atomoxetine	63	94	4	6.0	-	-	-	-
Risperidone	31	46.3	36	53.7	-	-	-	-
Aripiprazole	61	91.0	6	9.0	-	-	-	-
Clonidine	56	83.6	11	16.4	-	-	-	-
Anticonvulsant	27	40.3	40	59.7	-	-	-	-
Number of psychotropic medications	27	40.3	40	59.7	27	40.3	40	59.7

## Discussion

In this study, one of the main findings was that of the 67 children and adolescents with ASD accessing psychiatric care, most were males (60 males; 89.6%), this being consistent with the literature.^26,27,28,29,30,31^ The literature has reported that male children with ASD display more externalising behaviours than their female counterparts such as aggression and hyperactivity,^32^ which may explain an earlier presentation to psychiatric services. Other possibilities for the male predominance may include sex-linked genetic and hormonal factors that are thought to contribute to the risk of developing ASD, for example, prenatal hormones could diminish risk in female children while promoting it in males.^33^ The female protective model posits that a higher threshold of genetic liability is necessary for females to develop ASD versus males.^34^ The ‘extreme male brain theory’ also attempts to explain this gender bias – it suggests that foetal testosterone exposure may be the underlying gender difference in autistic traits.^35^

In terms of the racial demographics of this study, the bulk of participants were black children which is not consistent with international findings. The USA Centers for Disease Control and Prevention (CDC) community report of 2020 on ASD reports that black and Hispanic children are less likely to be identified with ASD than white children.^36^ Our results, however, are a reflection of the demographics of KwaZulu-Natal which has a black population of 87%.^37^

Fifteen individuals had a positive family history of mental illness in a first degree relative; however, the nature of the mental illness was not available in the medical records. This information is important in the context of ASD which is thought to be a highly heritable condition with a pronounced genetic contribution, reinforced by twin studies showing prominent concordance among monozygotic twins.^38^ Studies have demonstrated that ASD clusters in families, and estimate the quantity of phenotype variance because of heritability to be approximately 90%.^39^ Psychiatric disorders are more frequent among the relatives of children with ASD.^40^ Larsson et al. in a Danish study in 2005 showed that parental schizophrenia, affective disorders and substance use disorders were associated with ASD.^41^ These findings were echoed by Daniels et al.^42^ and Jokiranta et al.^43^ In a study in Taiwan in 2022, it was shown that patients with ASD are more likely to have parents with psychiatric disorders such as schizophrenia and psychotic disorders, mood, anxiety and personality disorders.^44^ In addition, maternal psychiatric diagnosis appeared to have a more profound effect on outcome than paternal diagnosis.^44^

In terms of their clinical variables, there was a delay between the recognition of first symptoms and a formal ASD diagnosis of approximately 3 years. The most common initial reason for referral to psychiatry being behavioural problems followed by speech delay. It should be noted that the early warning signs of possible ASD include poor joint attention, diminished eye contact, a lack of orienting to verbal call, decreased facial expression and social smile, and a lack of or poor quality of movements.^29^ In addition, the onset may be characterised by regression of language and social interaction between 16 and 20 months of age^45^ or sometimes by psychomotor delay or even by developmental stagnation.^46^

The delay in diagnosis of ASD from first symptom recognition to a definitive diagnosis of ASD is concerning, as early diagnosis and prompt intervention for ASD and associated comorbidities is critical for a number of reasons. Between the ages of birth and 6 years, the brain is geared to acquire social and verbal skills.^47^ Initiating and teaching such skills when the brain expects to develop these skills is related to faster and more sustained responses than if they are taught at a later stage.^48^ Furthermore, early detection and intervention offers the opportunity to empower families and caregivers for long-term advocacy and assistance of their children.^49^ Lastly, timeous diagnosis allows for monitoring and early intervention for co-occurring conditions such as ADHD, speech and sleep and eating problems.^50^ Early interventions which are both high in quality and intensity will result in increases in the individual’s intellectual, social, speech and adaptive behaviour, and decreases in life-long costs of special education, sheltered living and supported employment.^51^

The delay in diagnosis may be associated with multiple factors, such as in the USA CDC community report of 2023 on ASD, which showed that stigma, a lack of access to healthcare services because of non-citizenship or low income and non-English primary language are potential barriers to identification of children with ASD, especially among Hispanic children.^52^ The challenge of late diagnosis and role of social and cultural stigma were also noted in a review of ASD in Africa, and the authors of this review further noted that other important factors influencing delay in diagnosing ASD in the African setting may be a lack of specialised healthcare professionals and services, and a lack of awareness and understanding of ASD among healthcare service providers.^53^ Late identification and diagnosis may be further attributed to a lack of knowledge in parents pertaining to normal developmental milestones.^54^ In addition, they may also have a limited understanding of developmental disorders such as ASD^45^ or may be in denial about their child’s condition. Impediments to diagnosing and managing ASD include limited experience, inadequate educational resources, a lack of referral centres, failure of family support systems, deficient specialists in developmental disorders, scarce therapists, and insufficient time for a thorough assessment.^55^

In addition, Mazurek et al. report that older current age, lower socio-economic circumstances, higher IQ, and lower levels of autism symptoms are associated with later age of initial diagnosis. Lower parental education was also significantly associated with delays in diagnosis. They also found that children with greater current behavioural symptoms, and those with a previous psychiatric disorder diagnosis were diagnosed later with ASD than those without psychiatric diagnoses.^56^ Finally, importantly in low- and middle-income settings, health system factors which delay diagnosis include a backlog of patients to be seen^48^ and a lack of qualified healthcare providers^57^ may result in delay in diagnosis. In South Africa, families with a child with ASD may experience marked difficulties in gaining access to diagnostic, intervention, and educational services.^58,59^

In terms of ASD characteristics, the commonest presenting features reported in this study were a lack of reciprocal social interaction (100%), stereotypic movements (85.1%), language delay (74.6%) and hyperactivity (68.7%), this being similar to other studies.^60,61^ In a study conducted by Malhi et al., of the 33 patients with ASD, no peer play was found in 95.2% of children and motor stereotypies were also present in 85.7%.^60^ In another cross-sectional observational study of 112 children with ASD, the authors found a lack of reciprocal social interaction in 80.49%, language delay in 85.37%, and stereotypic movements in 53.66% as the most common features.^61^

Comorbid ADHD was noted in 55.2% (*n* = 37) and intellectual disability in 50.7% (*n* = 34). Comorbidity is the co-occurrence of two or more disorders in the same individual.^62^ Youth with ASD suffer from a significantly greater number of comorbid conditions than youth without ASD.^63^ Simonoff et al. (2008) report that 70.0% of children with ASD had at least one comorbid disorder and 41.0% had two or more conditions.^64^ Bradley and colleagues note that individuals with ASD showed four times more psychiatric comorbidity than those without ASD.^65^ Fodstad et al. noted that there was an increasing trend of comorbid symptoms as the child grew older.^66^ Mannion et al. also found that 46.1% of children and adolescents with ASD had a comorbid disorder, and when intellectual disability was included, this increased to 78.7%.^67^ These findings are consistent with this study also, where 50.7% of patients were diagnosed with comorbid intellectual disability.

The prevalence of ADHD in those living with ASD has ranged from 14% to 78%.^68^ Mannion et al. found that 18% of children and adolescents with ASD had a comorbid diagnosis of ADHD.^67^ Skokauskas and colleagues compared children with ASD to those without this diagnosis,^69^ and found that 44.78% of the ASD group met criteria for clinically significant ADHD. In this study, 37 of the 67 children and adolescents were diagnosed with ADHD (55.2%), which is in keeping with findings of another study at a paediatric neurology public hospital in KwaZulu-Natal, which reported a 57% prevalence rate of ADHD.^70^

The finding in this study that 29.9% of patients had a comorbid diagnosis of epilepsy is consistent with other studies such as 30.0% prevalence of comorbid seizure disorders in ASD in a study by Maniram et al. in 2023.^70^ Varying rates of epilepsy have been found and other studies have reported different rates; for example, Mannion et al. found that 10.1% of children and adolescents with ASD had a comorbid diagnosis of epilepsy.^67^ Epilepsy in ASD is associated with intellectual disability and the more severe the intellectual disability, the higher the prevalence of epilepsy.^71^ Bolton et al. also reported that 22% of individuals with ASD had epilepsy.^72^ Smith et al. report that those with ASD and a comorbid condition such as epilepsy were more impaired in terms of psychopathology than those with intellectual disability, epilepsy or ASD on its own.^73^ Hence, comorbid disorders may also have negative implications on outcome if not recognised and managed.

Regarding their management, non-pharmacological treatment modalities are critical for treating ASD and to assist with the core symptoms, they are easily accepted by parents and have fewer side effects.^74^ In this study, 45 (67.2%) patients were assessed by occupational therapy and 50 (74.6%) had an educational psychology assessment. However, only one-third (37%) had attended speech therapy, 10.4% were seen by the social worker and 11.9% by clinical psychology department, suggesting that care from the multi-disciplinary team may not be optimal, considering the high rates of comorbidities. Similarly, Maniram et al. report that less than half (44%) of patients were being seen by speech and/or occupational therapy, of whom, 23% were recommended by the prescriber of the pharmacological management.^70^

With regards to medication in this study, all those with ASD were on psychotropic medication and 40 patients (59.7%) were on more than one, the most widely used being anticonvulsants (59.7%), risperidone (53.7%), and methylphenidate (28.4%). This is consistent with a systematic literature review by Ritter et al., which found that most of the youth with ASD were taking two or more medications, psychotropic or non-psychotropic, simultaneously. The authors found that the rates of polypharmacy range across studies from 6.8% – 87% among autistic youth, and noted that having psychiatric comorbidities, self-injurious behaviours and physical aggression as well as being male and older were associated with higher rates of polypharmacy.^75^ Similarly, another recent study reported that 57% of patients with ASD were treated with polypharmacy of three or more medications and 83% were prescribed medications within a 1 year period of diagnosis and received a mean of four medications. It was evident that polypharmacy rates were higher in the ASD group compared to the general population.^76^ In a study of 33 565 children with ASD, Spencer et al. report that 35% of children had evidence of psychotropic polypharmacy. They found that older children and those with evidence of comorbid conditions such as epilepsy or ADHD had higher rates of psychotropic use and/or polypharmacy.^77^

In the other local study, the likelihood of psychotropic medication combination use among children with ASD was greatest among the 7–9 year age group, with males across all age groups requiring a combination of psychotropic medications more often than female patients. In contrast to this study, risperidone was used in 88% of patients, followed by methylphenidate in 32% and sodium valproate in only 16% of children,^70^ which may be because of their study being based in a paediatric clinic, which tends to focus on treating children less than 12 years of age.

## Limitations

This retrospective chart review was conducted at only two public sector psychiatric services in Durban, KwaZulu-Natal province, and did not include patients from the private health sector which may limit the generalisability of findings to the community at large. The sample size was small (67 patients), and there was missing information in some files because of incomplete documentation which were then excluded. Occasionally it was difficult to interpret information in the charts and a variance in the quality of information recorded by the medical professionals was noted. The period of analysis included the COVID-19 pandemic lockdown period, which may have limited help-seeking by caregivers and therefore affected the sample size. The timing of the study during the COVID-19 pandemic is a limitation as the pandemic influenced not only help-seeking by caregivers, but availability of health care professionals to assess and manage children with neurodevelopmental disorders particularly when mental health services were curtailed to support the COVID-19 crisis. It is recommended that additional studies review pathways to care after the pandemic. In addition, the use of pharmacotherapy may be artificially high in that patients are referred specifically to psychiatry for psychotropic medications as they may have not responded to other psychosocial treatments, their management status not reflecting patients who do not attend such a specialised facility. A final limitation is the lack of being able to assess use of alternative therapies.

## Recommendations

A diagnosis of ASD is often delayed and associated with comorbidity which may impact on outcome. Improved public health awareness is essential to reach parents, caregivers and teachers of children with ASD to ensure the early diagnosis and implementation of a multi-disciplinary approach to ensuring favourable outcomes. Moreover, we need to promote ASD in the curriculum of all health care workers to improve diagnosis. Research is needed at primary health care and community level to identify blockages in the system of having young children timeously referred for diagnosis and management.

Parents and caregivers must be empowered with knowledge about ASD so as to better relate to and understand their children.^49^ Increased awareness in parents and caregivers allows for greater support and advocacy for the special needs of their children.^49^ Early diagnosis enables close monitoring and intervention for associated comorbidities such as ADHD.^50^ Early detection improves outcomes as it allows for developmental trajectories be positively influenced in the phase of highest neuroplasticity (birth to 6 years of age).^47^

Standardised national policies which are evidence-based and in the best interest of individuals with ASD need to be formulated and implemented^78^ Swift detection and early intervention in ASD enhances long-term prognosis of the affected child and family, and decreases the financial burden on health care systems.

South Africa is known to have considerable health disparities.^78^ In order to enable access to comprehensive health care, outreach to disadvantaged communities is encouraged.^78^ Screening tools for ASD which are language-specific and culture-sensitive need to be formulated and implemented.^59^

Finally, parental education and training is the cornerstone of treatment of ASD. There is a lack of specialist service providers in most state facilities. By involving parents and caregivers, children with ASD can receive early interventions despite low resource settings.^78^

## Conclusion

This study echoes findings cited in existing literature regarding the clinical profile of children presenting with ASD. The strong male predilection of ASD coupled with significant comorbidities are in keeping with previous research. This study illustrates a persistent delay in diagnosis of approximately 3 years and low rates of allied health care involvement. It is important to recognise that prompt identification of and intervention in children with ASD capitalises on the period of highest neuroplasticity. In this way, the social, communication and language skills and functioning of those affected are improved, enhancing their quality of life and long-term prognosis.

## References

[1] American Psychiatric Association (APA). Diagnostic and Statistical Manual of mental disorders. 5th ed. Arlington, VA: American Psychiatric Association; 2013.

[2] Lord C, Elsabbagh M, Baird G, Veenstra-Vanderweele J. Autism spectrum disorder. Lancet. 2018;392(10146):508–520. 10.1016/S0140-6736(18)31129-2 30078460 PMC7398158

[3] Lord C, Brugha TS, Charman T, et al. Autism spectrum disorder. Nat Rev Dis Primers. 2020;6(1):5. 10.1038/s41572-019-0138-4 31949163 PMC8900942

[4] Divan G, Bhavnani S, Leadbitter K, et al. Annual research review: Achieving universal health coverage for young children with autism spectrum disorder in low- and middle-income countries: A review of reviews. J Child Psychol Psychiatry. 2021;62(5):514–535. 10.1111/jcpp.13404 33905120

[5] Buescher AV, Cidav Z, Knapp M, Mandell DS. Costs of autism spectrum disorders in the United Kingdom and the United States. JAMA Pediatr. 2014;168(8):721–772. 10.1001/jamapediatrics.2014.210 24911948

[6] Lemmi V, Knapp M, Ragan I. The autism dividend: Reaping the rewards of a better investment [homepage on the Internet]. 2017. Available from: https://nationalautistictaskforce.org.uk/wp-content/uploads/2020/02/autism-dividend-report.pdf

[7] World Health Assembly, Resolution 67.8. WHA 67.8/ Comprehensive and coordinated efforts for the management of autism spectrum disorders. UN and WHO Resolutions and Declarations. Resolutions and Declarations on Child and Youth, Resolutions and Declarations on Mental Health. May 24. Geneva: World Health Organization.

[8] Miller M, Iosif AM, Hill M, et al. Response to name in infants developing autism spectrum disorder: A prospective study. J Pediatr. 2017;183:141–146.e1. 10.1016/j.jpeds.2016.12.071 28162768 PMC5370582

[9] Zeidan J, Fombonne E, Scorah J, et al. Global prevalence of Autism: A systematic review update. Autism Res. 2022;15(5):778–790. 10.1002/aur.2696 35238171 PMC9310578

[10] Zwaigenbaum L, Bauman ML, Stone WL, et al. Early identification of autism spectrum disorder: Recommendations for practice and research. Pediatrics. 2015;136(Suppl 1):S10–S40. 10.1542/peds.2014-3667C 26430168 PMC9923897

[11] Xue Ming, Brimacombe M, Chaaban J, Zimmerman-Bier B, Wagner GC. Autism spectrum disorders: Concurrent clinical disorders. J Child Neurol. 2008;23(1):6–13. 10.1177/0883073807307102 18056691

[12] Collins H, Siegel M. Recognising and treating comorbid psychiatric disorders in people with autism spectrum disorder [homepage on the Internet]. Psychiatrictimes.com 29 August 2019. Maine: Maine Behavioural Healthcare. [cited 2023 Aug 20]. Available from: https://www.psychiatrictimes.com/view/recognising-and-treating-comorbidpsychiatric-disorders-people-autism;MaineBehaviouralHealthcare

[13] Hossain MM, Khan N, Sultana A, et al. Prevalence of comorbid psychiatric disorders among people with autism spectrum disorder: An umbrella review of systematic reviews and meta-analyses. Psychiatry Res. 2020;287:112922. 10.31124/advance.11497014 32203749

[14] Rong Y, Yang CJ, Jin Y, Wang Y. Prevalence of attention-deficit/hyperactivity disorder in individuals with autism spectrum disorder. Res Autism Spectrum Dis. 2021;83;101759. 10.1016/j.rasd.2021.101759

[15] Stevens T, Peng L, Barnard-Brak L. The comorbidity of ADHD in children with ASD. Res Autism Spectrum Dis. 2016;31:11–18. 10.1016/j.rasd.2016.07.003

[16] Van Steensel F, Bogels S, Perrin S. Anxiety disorders in children and adolescents with ASD: A meta-analysis. Psychol Rev. 2011;14:302–317. 10.1007/s10567-011-0097-0 PMC3162631 21735077

[17] Hollocks MJ, Lerh JW, Magiati I, Meiser-Stedman R, Brugha TS. Anxiety and depression in adults with autism spectrum disorder: A systematic review and meta-analysis. Psychol Med. 2019;49(4):559–572. 10.1017/S0033291718002283 30178724

[18] Hedley D, Uljarevic M, Foley KR, Richdale A, Trollor J. Risk and protective factors underlying depression and suicidal ideation in autism spectrum disorder. Depress Anxiety. 2018;35(7):648–657. 10.1002/da.22759 29659141

[19] Vannucchi G, Masi G, Toni C, Del’Osso L, Erfuth A, Perugi G. Bipolar disorder in adults with Asperger’s Syndrome: A systematic review. J Affect Disord. 2014;168:151–160. 10.1016/j.jad.2014.06.042 25046741

[20] Gotham K, Unruh K, Lord C. Depression and its measurement in verbal adolescents and adults woth autism spectrum disorder. Autism. 2015;19(4):491–504. 10.1177/1362361314536625 24916450 PMC4467786

[21] Kahl U, Schunke O, Schottle D, et al. Tic phenomenology and tic awareness in adults with autism. Mov Disord Clin Pract. 2015;2:237–242. 10.1002/mdc3.12154 30363532 PMC6178731

[22] Canitano R, Vivanti G. Tics and Tourette syndrome in autism spectrum disorder. Autism. 2007;11(1):19–28. 10.1177/1362361307070992 17175571

[23] Baron-Cohen S, Scahill VL, Izaguirre J, Hornsey H, Robertson MM. The prevalence of Gilles de la Tourette syndrome in children and adolescents with autism: A large-scale study. Psychol Med. 1999;29(5):1151–1159. 10.1017/S003329179900896X 10576307

[24] Kim YR, Song DY, Bong G, Han JH, Kim JH, Yoo HJ. Clinical characteristics of comorbid tic disorders in autism spectrum disorder: Exploratory analysis. Child Adolesc Psychiatry Ment Health. 2023;17(1):71. 10.1186/s13034-023-00625-8 37309007 PMC10262579

[25] Zwaigenbaum L, Bauman ML, Choueiri R, et al. Early Identification and interventions for autism spectrum disorder: Executive summary. Pediatrics. 2015;136(Suppl 1):S1–S9. 10.1542/peds.2014-3667B 26430167 PMC9923899

[26] Vargason T, Frye RE, McGuinness DL, et al. Clustering of co-occurring conditions in autism spectrum disorder during early childhood: A retrospective analysis of medical claims data. Autism Res. 2019;12(8):1272–1285. 10.1002/aur.2128 31149786 PMC6688922

[27] González-Cortés T, Gutiérrez-Contreras E, Espino-Silva PK, et al. Clinical profile of autism spectrum disorder in a pediatric population from Northern Mexico. J Autism Dev Disord. 2019;49(11):4409–4420. 10.1007/s10803-019-04154-2 31385173

[28] Kurasawa S, Tateyama K, Iwanaga R, Ohtoshi T, Nakatani K, Yokoi K. The Age at diagnosis of autism spectrum disorder in children in Japan. Int J Pediatr. 2018;2018:5374725. 10.1155/2018/5374725 29853922 PMC5964587

[29] Parmeggiani A, Corinaldesi A, Posar A. Early features of Autism spectrum disorder: A cross-sectional study. Ital J Pediatr. 2019;45(1):144. 10.1186/s13052-019-0733-8 31727176 PMC6857294

[30] Bhat BA, Hussain AM, Qadir W, et al. Clinico-socio demographic profile of children with autism spectrum disorders from a Tertiary Care Hospital in Kashmir, India. J Child Dev Disord. 2019;5(2):5. 10.36648/2472-1786.5.2.82

[31] Ramachandram S. Clinical characteristics and demographic profile of children with autism spectrum disorder (ASD) at child development clinic (CDC), Penang Hospital, Malaysia. Med J Malaysia. 2019;74(5):372–376. 31649211

[32] Bargiela S, Steward R, Mandy W. The experiences of late- diagnosed women with autism spectrum conditions: An investigation of the female autism phenotype. J Autism Dev Disord. 2016;46:3281–3294. 10.1007/s10803-016-2872-8 27457364 PMC5040731

[33] Singer L. Thoughts about sex and gender differences from the next generation of autism scientists. Mol Autism. 2015;6:52. 10.1186/s13229-015-0046-8 26388981 PMC4574348

[34] Robinson EB, Lichtenstein P, Anckarsater H, Happe F, Ronald A. Examining and interpreting the female protective effect against autistic behaviour. Proc Natl Acad Sci U S A. 2013;110(13):5258–5262. 10.1073/pnas.1211070110 23431162 PMC3612665

[35] Baron-Cohen S. The extreme male brain theory of autism. Trends Cogn Sci. 2003;6(6):248–254. 10.1016/S1364-6613(02)01904-6 12039606

[36] CDC Centre for Disease control and prevention. US Department of Health and Human services ASD homepage 2020 [homepage on the Internet] [cited 2023 June 26]. Available from: https://www.cdc.gov

[37] Statistics South Africa. Midyear population estimates [homepage on the Internet]. Pretoria: StatsSA; 2017. Available from: https://www.statssa.gov.za/publications/P0302/P03022017.pdf

[38] Folstein S, Rutter M. Infantile autism: A genetic study of 21 twin pairs. J Child Psychol Psychiatry. 1977;18(4):297–321. 10.1111/j.1469-7610.1977.tb00443.x 562353

[39] Sandin S, Lichtenstein P, Kuja-Halkola R, Hultman C, Larsson H, Reichenberg A. The heritability of autism spectrum disorder. JAMA. 2017;318(12):1182–1184. 10.1001/jama.2017.12141 28973605 PMC5818813

[40] Ingersoll B, Hambrick D. The relationship between broader autism phenotype, child severity, and stress and depression in parents of children with Autism spectrum disorders. Res Autism Spectrum Dis. 2011;5(1):337–344. 10.1016/j.rasd.2010.04.017

[41] Larsson HJ, Eaton WW, Madsen KM, et al. Risk factors for autism: Perinatal factors, parental psychiatric history and socioeconomic status. Am J Epidemiol. 2005;161(10):916–925 10.1093/aje/kwi123 15870155

[42] Daniels JL, Forssen U, Hultman CM, et al. Parental psychiatric disorders associated with autism spectrum disorders in the offspring. Paediatrics. 2008;121(5):1357–1362. 10.1542/peds.2007-2296 18450879

[43] Jokiranta E, Brown AS, Heinimaa M, Cheslack-Postava K, Suominen A, Sourander A. Parental psychiatric disorders and autism spectrum disorders. Psychiatry Res. 2013;207(3):203–211. 10.1016/j.psychres.2013.01.005 23391634 PMC3654001

[44] Yu T, Chang KC, Kuo PL. Paternal and maternal psychiatric disorders associated with offspring autism spectrum disorders: A case-control study. J Psychiatry Res. 2022;151:469–475. 10.1016/j.jpsychires.2022.05.009 35609363

[45] Ozonoff S, Iosif AM, Baguio F, et al. A prospective study of the emergence of early behavioral signs of autism. J Am Acad Child Adolesc Psychiatry. 2010;49(3):256–266.e1–2. 10.1016/j.jaac.2009.11.009 20410715 PMC2923050

[46] Siperstein R, Volkmar F. Brief report: Parental reporting of regression in children with pervasive developmental disorders. J Autism Dev Disord. 2004;34(6):731–734. 10.1007/s10803-004-5294-y 15679193

[47] Courchesne E, Campbell K, Solso S. Brain growth across the life span in autism: Age- specific changes in anatomical pathology. Brain Res. 2011;1380:138–145. 10.1016/j.brainres.2010.09.101 20920490 PMC4500507

[48] Huttonlocher P. Neural plasticity: The effects of environment on the development of cerebral cortex. Cambridge, MA: Harvard University Press; 2002.

[49] Rogers SJ, Dawson G, Vismara LA. An early start for your child with autism: Using everyday activities to help kids connect, communicate and learn. New York, NY: Guillford Press; 2012.

[50] Bauman ML. Medical comorbidities in autism: Challenges to diagnosis and treatment. Neurotherap J Am Soc Neurotherap. 2010;7(3):320–327. 10.1016/j.nurt.2010.06.001 PMC5084236 20643385

[51] Cidav Z, Munson J, Estes A, Dawson G, Rogers S, Mandell D. Cost offset associated with Early Start Denver Model for children with autism. J Am Acad Child Adolesc Psychiatry. 2017;56(9):777–783. 10.1016/j.jaac.2017.06.007 28838582 PMC7007927

[52] CDC Centre for Disease Control and Prevention. US Department of Health and Human services ASD homepage [homepage on the Internet] 2023 [cited 2023 July 7]. Available from: https://www.cdc.gov

[53] Aderinto N, Olatunji D, Idowu O. Autism in Africa: Prevalence, diagnosis, treatment and the impact of social and cultural factors on families and caregivers: A review. Ann Med Surg (Lond). 2023;85(9):4410–4416. 10.1097/MS9.0000000000001107 37663716 PMC10473371

[54] Van Biljon KA. Early identification of learners with ASD: Drawing on developmental histories. Early Child Dev Care. 2019;189(1):157–167. 10.1080/03004430.2017.1301934

[55] Matlou MJ. Screening, diagnosis and management of autism spectrum disorders amongst healthcare practitioners in South Africa [homepage on the Internet]. 2021. Available from: https://scholar.ufs.ac.za

[56] Mazurek MO, Handen BL, Wodka EL, Nowinski L, Butter E, Engelhardt CR. Age at first autism spectrum disorder diagnosis: The role of birth cohort, demographic factors, and clinical features. J Dev Behav Pediatr. 2014;35(9):561–569. 10.1097/DBP.0000000000000097 25211371

[57] Bisgaier J, Levinson D, Cutts DB, Rhodes KV. Access to Autism evaluation appointments with developmental-behavioral and neurodevelopmental subspecialists. Arch Pediatr Adolesc Med. 2011;165(7):673–674. 10.1001/archpediatrics.2011.90 21727283

[58] Basco WT, Rimsza ME. Committee on pediatric workforce-American Academy of Pediatrics. Pediatrician workforce policy statement. Pediatrics. 2013;132(2):390–397. 10.1542/peds.2013-1517 23897908

[59] De Vries PJ. Thinking globally to meet local needs: Autism spectrum disorders in Africa and other low-resource environments. Curr Opin Neurol. 2016;29(2):130–136. 10.1097/WCO.0000000000000297 26886354

[60] Malhi P, Singhi P. A retrospective study of toddlers with autism spectrum disorder: Clinical and developmental profile. Ann Indian Acad Neurol. 2014;17(1):25–29. 10.4103/0972-2327.128537 24753655 PMC3992765

[61] Kondekar A, Joshi S, Shah H, Subramanyam A. Clinical profile of children with autism spectrum disorder in tertiary care centre. Int J Contemp Pediatr. 2016;3(2):334–9. 10.18203/2349-3291.ijcp20160947

[62] Matson JL, Nebel-Schwalm MS. Comorbid psychopathology with autism spectrum disorder in children: An overview. Res Dev Disabil. 2007;28(4):341–352. 10.1016/j.ridd.2005.12.004 16765022

[63] Joshi G, Petty C, Wozniak J, et al. The heavy burden of psychiatric comorbidity in youth with autism spectrum disorders: A large comparative study of a psychiatrically referred population. J Autism Dev Disord. 2010;40(11):1361–1370. 10.1007/s10803-010-0996-9 20309621

[64] Simonoff E, Pickles A, Charman T, et al. Psychiatric disorders in children with autism spectrum disorders: Prevalence, comorbidity, and associated factors in a population-derived sample. J Am Acad Child Adolesc Psychiatry. 2008; 47(8):921–929. 10.1097/CHI.0b013e318179964f 18645422

[65] Bradley EA, Summers JA, Wood HL, Bryson SE. Comparing rates of psychiatric and behavior disorders in adolescents and young adults with severe intellectual disability with and without Autism. J Autism Dev Disord. 2004;34(2):151–161. 10.1023/B:JADD.0000022606.97580.19 15162934

[66] Fodstad J, Rojahn J, Matson J. Emergent comorbidity in at risk children with and without autism spectrum disorder – A cross-sectional study. J Dev Phys Disabil. 2010;22;381–400. 10.1007/s10882-010-9202-4

[67] Mannion A, Leader G. Comorbidity in autism spectrum disorder: A literature review. Res Autism Spectrum Dis. 2013;7(2):1595–1616. 10.1016/j.rasd.2013.09.006

[68] Gargaro BA, Rinehart NJ, Bradshaw JL, Tonge BJ, Sheppard DM. Autism and ADHD: How far have we come in the comorbidity debate? Neurosci Biobehav Rev. 2011;35:1081–1088. 10.1016/j.neubiorev.2010.11.002 21093480

[69] Skokauskas N, Gallagher L. Mental health aspects of autism spectrum disorder in children. J Intellect Disabil Res. 2012;56:248–257. 10.1111/j.1365-2788.2011.01423.x 21554467

[70] Maniram J, Oosthuizen F, Karrim SB. An overview of pharmacotherapy in the management of children with ASD at a public hospital in Kwazulu-Natal. Child Psychiatry Hum Dev. 2023. 10.1007/s10578-023-01514-z PMC11485179 36944778

[71] Amiet C, Gourfinkel-An I, Bouzamondo A, et al. Epilepsy in autism is associated with intellectual disability and gender: Evidence from a meta-analysis. Biol Psychiatry. 2008;64(7):577–582. 10.1016/j.biopsych.2008.04.030 18565495

[72] Bolton PF, Carcani-Rathwell I, Hutton J, Goode S, Howlin P, Rutter M. Epilepsy in autism: Features and correlates. Br J Psychiatry. 2011;198(4):289–294. 10.1192/bjp.bp.109.076877 21972278 PMC3065774

[73] Smith KRM, Matson JI. Psychopathology: Differences among adults with intellectually disabled comorbid autism spectrum disorders and epilepsy. Res Dev Disabil. 2010b;31:743–749. 10.1016/j.ridd.2010.01.016 20206472

[74] Jiang X, Song M, Qin W, Xiao J, Xu X, Yuan Q. Non-pharmaceutical therapy for autism spectrum disorder: A Protocol for systematic review and network meta-analysis. Medicine (United States). 2022;101(7):E28811. 10.1097/MD.0000000000028811 PMC9281987 35363171

[75] Ritter C, Hewitt K, McMorris CA. Psychotropic polypharmacy among children and youth with Autism: A systematic review. J Child Adolesc Psychopharmacol. 2021;31(4):244–258. 10.1089/cap.2020.0110 33970024

[76] Bowden N, Thabrew H, Kokaua J, Braund R. National prescribing rates and polypharmacy for children and young people in New Zealand with and without ASD. Res ASD. 2020;78:101642. 10.1016/j.rasd.2020.101642

[77] Spencer D, Marshall J, Post B, et al. Psychotropic medication use and polypharmacy in children with autism spectrum disorders. Paediatrics. 2013;132(5):833–840. 10.1542/peds.2012-3774 PMC3813388 24144704

[78] Franz L, Adewumi K, Chambers N, Viljoen M, Baumgartner JN, de Vries PJ. Providing early detection and early intervention for autism spectrum disorder in South Africa: Stakeholder perspectives from the Western Cape province. J Child Adolesc Ment Health. 2018;30(3):149–165. 10.2989/17280583.2018.1525386 30403918 PMC6301128

